# The Validity of Surrogate Endpoints in Sub Groups of Metastatic Colorectal Cancer Patients Defined by Treatment Class and KRAS Status

**DOI:** 10.3390/cancers14215391

**Published:** 2022-11-01

**Authors:** Heather Poad, Sam Khan, Lorna Wheaton, Anne Thomas, Michael Sweeting, Sylwia Bujkiewicz

**Affiliations:** 1 Biostatistics Research Group, Department of Health Sciences, University of Leicester, Leicester LE1 7RH, UK; 2 Leicester Cancer Research Centre, University of Leicester, Leicester LE1 7RH, UK

**Keywords:** metastatic colorectal cancer, surrogate endpoints, bayesian meta-analysis, health technology assessment

## Abstract

**Simple Summary:**

When evaluating new cancer therapies in clinical trials, it may take a long time to estimate their effectiveness on overall survival, an outcome typically of main interest to regulatory decision-makers. To expedite access to new therapies for patients, regulatory agencies often make their decisions based on treatment effectiveness measured on surrogate outcomes; for example looking at the impact of treatment on delaying cancer recurrence, which can be measured earlier. For such decisions to be robust, a surrogate endpoint needs to be a valid predictor of overall survival. The validation can be complex and previous research in advanced colorectal cancer has suggested that the validity of a surrogate endpoint may depend on treatment class. We have investigated this and our results indicated that the validity of surrogate endpoints is stronger within some treatment classes compared to when ignoring the treatment class. Surrogate’s validity needs careful consideration to ensure appropriate regulatory decisions.

**Abstract:**

**Background and Aim:** Findings from the literature suggest that the validity of surrogate endpoints in metastatic colorectal cancer (mCRC) may depend on a treatments’ mechanism of action. We explore this and the impact of Kirsten rat sarcoma (KRAS) status on surrogacy patterns in mCRC. **Methods:** A systematic review was undertaken to identify randomized controlled trials (RCTs) for pharmacological therapies in mCRC. Bayesian meta-analytic methods for surrogate endpoint evaluation were used to evaluate surrogate relationships across all RCTs, by KRAS status and treatment class. Surrogate endpoints explored were progression free survival (PFS) as a surrogate endpoint for overall survival (OS), and tumour response (TR) as a surrogate for PFS and OS. **Results:** 66 RCTs were identified from the systematic review. PFS showed a strong surrogate relationship with OS across all data and in subgroups by KRAS status. The relationship appeared stronger within individual treatment classes compared to the overall analysis. The TR-PFS and TR-OS relationships were found to be weak overall but stronger within the Epidermal Growth Factor Receptor + Chemotherapy (EGFR + Chemo) treatment class; both overall and in the wild type (WT) patients for TR-PFS, but not in patients with the mutant (MT) KRAS status where data were limited. **Conclusions:** PFS appeared to be a good surrogate endpoint for OS. TR showed a moderate surrogate relationship with PFS and OS for the EGFR + Chemo treatment class. There was some evidence of impact of the mechanism of action on the strength of the surrogacy patterns in mCRC, but little evidence of the impact of KRAS status on the validity of surrogate endpoints.

## 1. Introduction

Metastatic colorectal cancer (mCRC) is an area in which targeted treatments have proven successful, with cetuximab and panitumumab being offered as first line treatment [1]. When evaluating novel cancer therapies in randomised controlled trials (RCTs), data on overall survival (OS) is of primary interest to regulatory and reimbursement decision-makers. However, the more successful the treatment, the longer the wait for sufficiently mature effectiveness data for OS. In such circumstances, to expedite access to new therapies to patients, a surrogate endpoint, such as tumour response (TR) or progression-free survival (PFS), may be used to determine the efficacy of the drug, for example at the regulatory decision stage, and re-evaluated when more mature OS data become available [2,3,4]. It is therefore important that surrogate endpoints are appropriately validated to ensure that they are good predictors of clinical benefit [5].

Historically, surrogate endpoint validation has been conducted based on data from RCTs of all therapies in a given disease area. With an improved understanding of cancer biology, targeted treatments are available to subgroups of patients often with specific biomarkers. This raises the question whether validity of a putative surrogate endpoint depends on the treatments’ mechanism of action. Buyse et al. [6] concluded that PFS can be used as a surrogate for OS for mCRC using data from trials comparing fluorouracil plus leucovorin with fluorouracil alone, raltitrexed, irinotecan and oxaliplatin. Subsequently, Giessen et al. [7] evaluated PFS as a surrogate endpoint for OS in mCRC, exploring surrogacy patterns in subgroups of RCTs defined by treatment classes including chemotherapy (Chemo) regimens and targeted therapies with anti-Vascular Endothelial Growth Factor (anti-VEGF) or anti-Endothelial Growth Factor Receptor (anti-EGFR) directed monoclonal antibodies. They concluded that for chemotherapy, PFS was an appropriate surrogate endpoint for OS, but for the targeted treatments explored, there was not enough RCT data available to make a conclusion with certainty. Most recently, Ciani et al. [8] explored PFS, TR and time to progression (TTP) as surrogate endpoints for OS in mCRC patients using data from RCTs of a broad range of pharmacological therapies. They concluded none of the putative surrogate endpoints had a particularly strong relationship with OS and suggested that the stronger surrogacy patterns seen previously may only apply to certain treatments or treatment classes as they may depend on treatments’ mechanism of action. In this paper, we investigate whether the validity of surrogate endpoints in mCRC depends on the mechanism of action of a treatment. We also explore whether surrogacy patterns depend on the patients’ status for the Kirsten rat sarcoma (KRAS); KRAS wild-type (WT) or KRAS mutant (MT) [9,10]. This biomarker has proven crucial in determining the response to anti-epidermal growth factor receptor (EGFR) targeted therapies [10], with the therapies performing well in the WT population. Recent meta-analysis have shown mutation status and tumour sidedness may impact survival and disease progression; KRAS mutations present in 35% of left sided and 46% of right sided tumours. Notably there are few studies reporting OS or PFS based on both of these variables [11]. Therefore it is plausible that KRAS status may also determine whether the surrogate endpoint is in the causal pathway of the disease process, and the extent to which the intervention effect is mediated through the surrogate.This assessment is important clinically, given the large proportion of patients who harbour WT tumours and are therefore eligible for EGFR based therapies.

We consider the validity of putative surrogate endpoints; PFS for OS, and TR for PFS or OS. We conducted a systematic review to identify all RCTs of pharmacological therapies in mCRC from January 2003 to April 2020 reporting treatment effects on the endpoints of interest. Subsequently, we investigated surrogacy patterns overall, in patient populations defined by KRAS status and whether the surrogacy patterns differed depending on treatment class.

## 2. Methods

### 2.1. Trial Identification

A systematic review was undertaken to identify all RCTs for pharmacological therapies in mCRC (PROSPERO ID: CRD42020167075 [12]). Three databases were reviewed, Embase [13], Medline [14] and Cochrane CENTRAL [15]. Papers published January 2003 onwards were searched. No restrictions were placed on language. Searches were carried out on 3 April 2020. Full search strategies are included in Appendix A.1.

RCTs reporting the effectiveness of therapies based on KRAS status were selected. Trials were included if they were RCTs comparing pharmacological treatments in mCRC patients and reported treatment effects on at least two of the three outcomes of interest (OS, PFS, TR). Trials had to report treatment effects for WT or MT KRAS status patient groups, or both. Trials were excluded if either arm included radiotherapy or surgery alone or in combination with a pharmacological treatment. Trials for biosimilar drugs were excluded. Titles and abstracts were screened independently by three reviewers (HP, SB, MS) until 95% agreement was reached for 10% of papers. One reviewer (HP) completed the remainder of title and abstract screening. Papers were then grouped by trial and reviewed as trials at the full text stage in a similar fashion to title and abstract review process.

### 2.2. Data Extraction

The following general study information was extracted from all eligible RCTs: author, title, year and journal of publication, definition of disease progression used, country(s) the RCT took place in, key inclusion and exclusion criteria, length of follow up, line of treatment and pharmacological treatments given in each arm. Treatments were classified by each drug’s mechanism of action (e.g., EGFR, VEGF, or EGFR + VEGF). Trials were grouped into treatment classes based on the mechanism of action of their experimental arm.

From the selected RCTs, data were collected on the treatment effects on TR, PFS and OS. Definitions used for each treatment effect are outlined in Appendix A.2. Treatment effects on PFS and OS were recorded as hazard ratios (HRs) with 95% Confidence Intervals (CIs) or *p*-values if no CIs were reported. One reviewer extracted data (HP) and 10% of the data extraction was reviewed independently by one other reviewer (LW). A risk of bias assessment was performed using a modified version of the Cochrane Risk of Bias tool [16].

Trial identification and data extraction was carried out using the systematic review software tools Covidence and EndNote.

### 2.3. Statistical Methods

The meta-analytic method by Daniels and Hughes [17] was used to evaluate the surrogate relationships across trials for each pair of outcomes of interest; (1) PFS as a surrogate for OS, (2) TR for PFS, and (3) TR for OS. The Daniels and Hughes approach uses Bayesian meta-regression to model the relationship between the treatment effects on the two outcomes (for example log HRs on PFS and OS), whilst taking into account the uncertainty around the effects on both outcomes and the correlation between them. The model was further applied separately to subgroups of patients defined by KRAS status.

To evaluate surrogate endpoints according to the mechanism of action jointly across treatment classes (either for the whole patient population or for KRAS sub-populations), the hierarchical method proposed by Papanikos et al. [18] was used, allowing for partial exchangeability. The method, extending the approach by Daniels and Hughes, allows for borrowing of information about the surrogacy patterns across treatment classes, which is particularly useful when the number of studies for some of the classes is small.

The surrogacy criteria outlined by Daniels and Hughes [17] were used to assess the strength of the surrogate relationships. The criteria state that a perfect surrogate relationship is defined by a regression line with intercept equal to zero (to ensure no effect on the surrogate endpoint implies no effect on the final outcome), a non-zero slope (ensuring the association between the treatment effects on the surrogate endpoint and final outcome), and zero conditional variance (ensuring a perfect prediction of the treatment effect on the final outcome is made based on the treatment effect on the surrogate endpoint). In practice, we consider a surrogate relationship strong if all the following is true: the 95% interval for the intercept includes zero, the 95% interval for the slope does not include zero and the conditional variance along with its upper interval is small. When exploring surrogacy patterns in subgroups defined by treatment class or KRAS status, we use these criteria to identify any groups where surrogacy may be stronger. In this Bayesian framework, we focus on uncertainty around these parameters rather than performing any hypothesis testing. A “take-one-out” cross-validation procedure was performed to investigate the predictive value of a putative surrogate endpoint [17]. A summary of the cross-validation procedure, and further statistical methods are included in Appendix A.3.

A Bayesian approach was used for the analyses performed in WinBUGS version 1.4.3. Analyses used 125,000 Markov chain Monte Carlo (MCMC) iterations including a 25,000 burn-in. Results are presented as a mean and 95% credible interval (CrI) for each of the parameters for surrogacy criteria. Data management and additional analyses were carried out using R version 4.1.0.

## 3. Results

### 3.1. Summary of Included Trials

**Figure 1:**
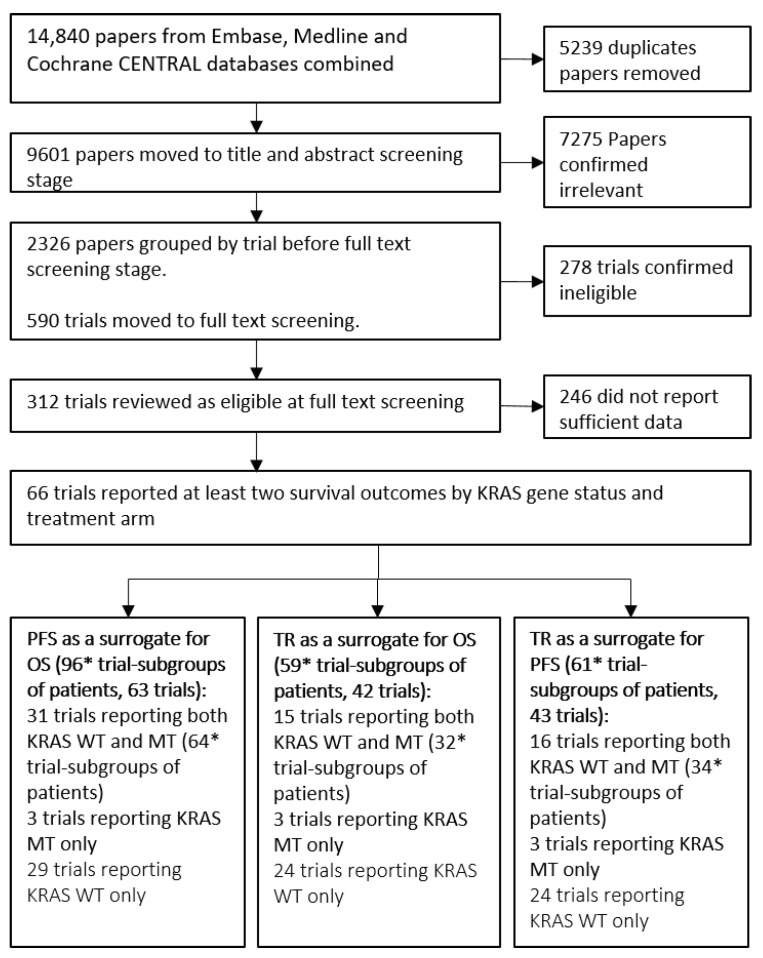
Flow diagram of the systematic review process. Groups of patients are either Kirstan rat sarcoma (KRAS) wild-type (WT) or KRAS mutant (MT) patients from a trial. PFS = Progression Free Survival, OS = Overall Survival, TR = Tumour Response. * 2 trials were multi-arm trials and reported two randomised treatment contrasts for each KRAS gene status.

Throughout the rest of this paper we refer to “trial-subgroups” rather than trials. This is to reflect that data included in the meta-analysis is at the subgroup level; for example, two subgroups from a single trial reporting treatment effects for KRAS WT and KRAS MT are entered separately. The systematic review process, outlined in Figure 1, identified 66 trials consisting of 100 trial-subgroups that reported sufficient information to be included. The trials investigated a broad range of treatments including Chemo, EGFR and VEGF therapies. The list of treatments and classifications for the analysis are included in Table 1. Seven treatment classes were defined for the analyses investigating the impact of the mechanism of action on surrogacy patterns. Fifteen of the trials had treatment arm comparisons that were unique and therefore were not grouped into a treatment class.

The flow chart in Figure 1 shows that 96 trial-subgroups (63 trials) were available for the evaluation of PFS as a surrogate for OS, 59 trial-subgroups (42 trials) for the analysis of TR as a surrogate for OS and 61 trial-subgroups (43 trials) for the evaluation of TR as a surrogate for PFS. A full list of trials included for the evaluation of each surrogate relationship is included in Table 2.

### 3.2. Exploration of Surrogate Relationships

We focus here on the results for PFS as a putative surrogate endpoint for OS and TR as surrogate for PFS; both overall and according to KRAS status or treatment class. The results and conclusions for TR as a surrogate endpoint for OS can be found in Appendix B.1.

#### 3.2.1. Surrogate Relationships Overall and by KRAS Status

**Figure 2:**
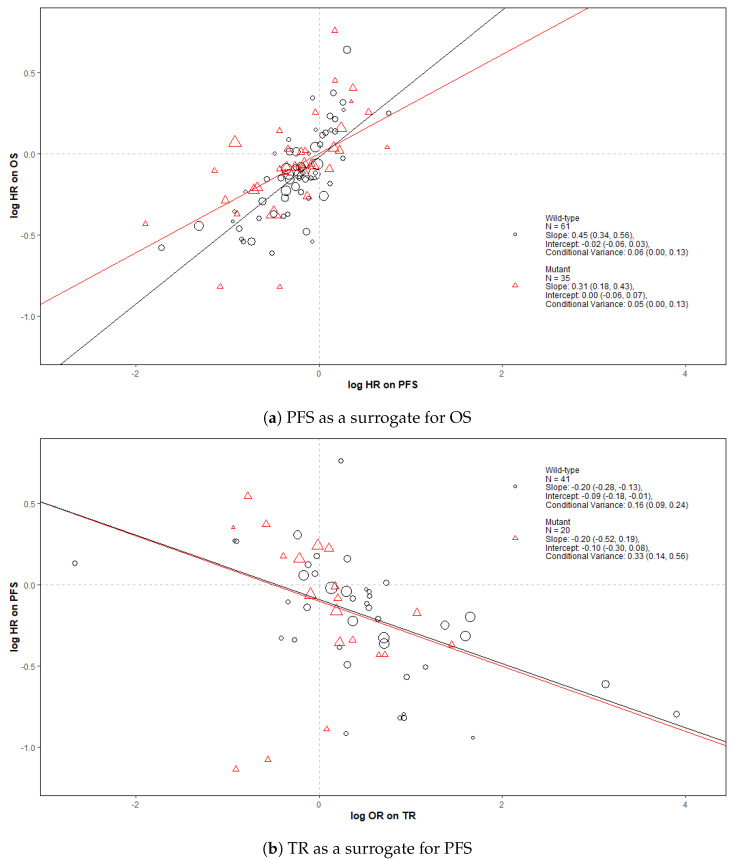
Bubble plots of the surrogate relationships in trial-subgroups of patients with KRAS WT and MT metastatic colorectal cancer (mCRC). The Slope, Intercept and Conditional variance are mean estimates with 95% Credible Intervals obtained from Daniels and Hughes model. N represents the number of trial-subgroups. HR = Hazard Ratio, OR = Odds Ratio.

**Figure 3:**
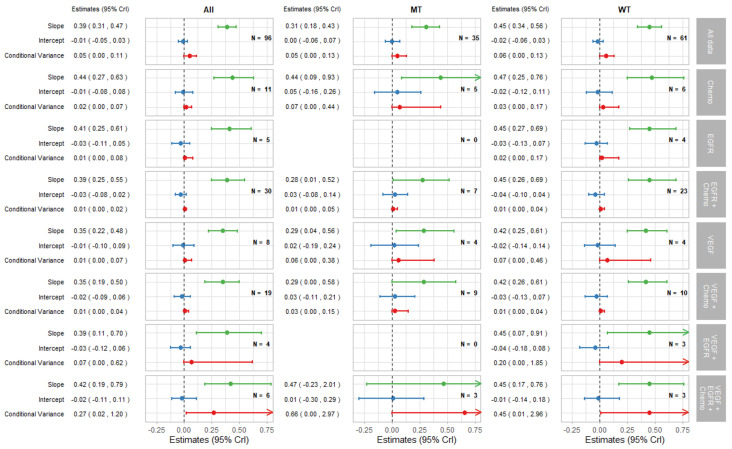
Forest plot of estimates of slope (green), intercept (blue), and conditional variance (red) for PFS as a surrogate for OS. N represents the number of trial-subgroups. CrI = Credible Interval.

**Figure 4:**
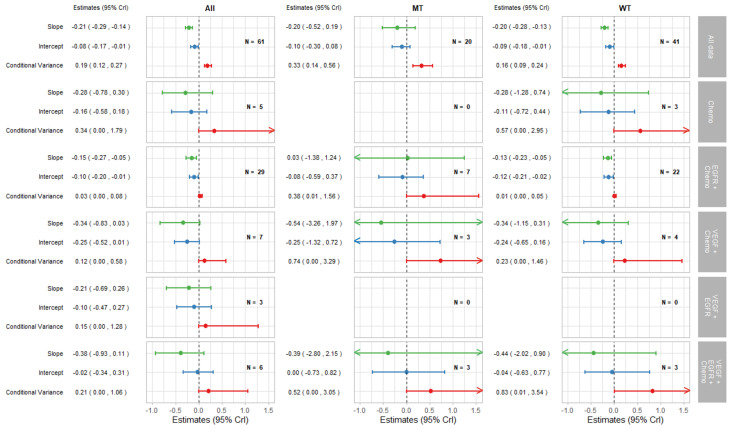
Forest plot of estimates of slope (green), intercept (blue), and conditional variance (red) for TR as a surrogate for PFS. N represents the number of trial-subgroups.

Figure 2 shows bubble plots representing data from all trial-subgroups included in the analysis, colour-coded by the KRAS status. The regression lines represent surrogate relationships by KRAS status, irrespective of treatment class, for each surrogate relationship. Surrogacy criteria for each pair of outcomes (both for all patients and KRAS subgroups) are represented in the top panels of Figure 3 and Figure 4 (marked “All data”), which correspond to the overall analysis marked by ‘All’ and the KRAS status subgroups marked by ‘MT’ and ‘WT’. Overall, the surrogacy was found to be strong for the PFS-OS surrogate relationship (Figure 2a and Figure 3). TR proved to be a sub-optimal surrogate endpoint for PFS, as indicated by a relatively large conditional variance as shown in Figure 2b and Figure 4.

The surrogate relationships between PFS and OS did not differ across KRAS subgroups where it was similar to the relationship in the overall cohort of patients, as can be seen in Figure 2a and Figure 3. For TR-PFS, the surrogacy pattern for KRAS WT was similar to the relationship for All data, as shown in the top panels of Figure 2b and Figure 4). However, the conditional variance was higher for the KRAS MT trial-subgroups where also the interval for the slope included zero, thus suggesting a weaker surrogate relationship compared to the KRAS WT and overall cohorts of patients.

#### 3.2.2. Surrogate Relationships by Treatment Class: Overall and in KRAS Subgroups

The remaining parts of the forest plots in Figure 3 and Figure 4 correspond to the surrogate relationships across treatment classes. To investigate any impact of the KRAS status on surrogacy patterns within the treatment classes, all results are presented for subgroups of patients according to the KRAS status as well as for all patients (WT and MT combined). The left, middle and right columns correspond to ‘All’ (for WT and MT combined), MT trial-subgroups and WT trial-subgroups, respectively. Each column shows the overall results at the top, discussed in the previous section, followed by the results for each treatment class including the intercepts, slopes and conditional variances.

Figure 3 shows results for surrogacy patterns between the treatment effect on PFS and OS. For the All trial-subgroups analysis, there were no distinct differences in surrogacy patterns between the treatment classes. The strong surrogate relationship seen for all of the data (represented in Figure 2a and the top row of Figure 3) holds for the individual treatment classes (with the exception for those classes with small numbers of trial-subgroups). However, the surrogate relationships appeared stronger, in terms of the smaller conditional variance, within most of the individual treatment classes (apart from VEGF + EGFR and VEGF + EGFR + Chemo) compared to the analysis including All data. This was also the case for the EGFR + Chemo treatment class for both MT and WT trial-subgroups and for VEGF + Chemo for the WT trial-subgroup only.

Figure 4 shows the results for TR-PFS surrogacy patterns. The surrogacy pattern was stronger within the EGFR + Chemo treatment class with the conditional variance of 0.03 (0.00, 0.08), which was much smaller compared to the conditional variance obtained from the analysis of all data; 0.19 (0.12, 0.27). The surrogacy criteria were not fully satisfied for the EGFR + Chemo treatment class, with the CrIs for the intercept not including zero for the analysis of All data and for the WT trial-subgroups alone. For the MT trial-subgroups, the TR-PFS surrogate relationship was weak. The trial-subgroups for the EGFR + Chemo treatment class contributed the majority of the data for each analysis, with little or no studies in other treatment classes; therefore, our inferences about the other treatment classes are limited. However, the change of the results from overall analysis of all data to those using data from the EGFR + Chemo treatment class suggests the importance of the mechanism of action in this surrogate relationship, which was true for both the overall result and KRAS WT subgroup of the population.

Cross-validation results for the surrogate relationship between PFS and OS are presented in Appendix B.2. In summary, treatment effect on PFS was a good predictor of the treatment effect on OS overall and within subgroups of treatment classes and KRAS status.

## 4. Discussion

Overall, our analyses showed that there was a strong surrogate relationship between the treatment effect on PFS and OS for mCRC, which supports existing knowledge in this area [6,7]. However the findings are stronger than the conclusions of Ciani et al. [8], who found that overall the surrogate relationship was sub-optimal; however, the criteria used for assessing surrogacy patterns differed. When considering solely bevacizumab and chemotherapy in the first and second line setting PFS was determined to be a good candidate as a surrogate endpoint for OS in patients with mCRC [85], however others have reported OS to be the preferred primary endpoint in the second line treatment of mCRC [86]. Furthermore, exploring the relationship by treatment class suggested some evidence that the mechanism of action may contribute to the strength of surrogacy patterns in mCRC for PFS-OS, as evidenced by smaller conditional variances within the treatment classes (with zero variance indicating a perfect association).

We found that overall the surrogate relationship between the treatment effects on TR and the effects on PFS or OS was weak for mCRC. However, there was some evidence that the surrogacy patterns may vary according to the mechanism of action.For EGFR+Chemo treatment class, the surrogacy pattern between TR and PFS was relatively strong except for the intercept not being zero; however, the upper interval for the intercept was close to zero. For TR-PFS pair of outcomes the results indicated some limited evidence that there is a difference in surrogacy between KRAS subgroups of patients, with MT trial-subgroup analyses showing weaker surrogacy than the WT trial-subgroup or the All data analyses.

Additional areas to consider when evaluating surrogate endpoints in mCRC include BRAF status. This is particularly important as individuals who harbour a BRAF V600 mutation often have greater risk of recurrence and poorer prognosis than patients who do not. There is improved overall survival with combination treatment of anti-EGFR and BRAF inhibitor treatment in these patients [87], which may impact the strength of a surrogate relationship. Further analysis such as side of tumour (left or right) or evidence of a PIK3CA mutation may be helpful, however this is often not reported.

## 5. Conclusions

This is the first review and meta-analysis investigating surrogacy patterns based on the KRAS status of patients and differentiating surrogacy patterns according to treatment class for mCRC patients. In summary, our results showed that PFS is a good surrogate for OS when evaluating pharmacological therapies for mCRC patients. The surrogate relationships between TR and PFS or OS, however, were found to be weak overall. There was evidence that the mechanism of action may contribute to the strength of surrogacy patterns in mCRC for PFS as a surrogate for OS as well as TR for PFS. These conclusions remained the same for the subgroups of patients according to their KRAS status.

## Figures and Tables

**Table 1:** Summary of treatment arm combinations of included trials within each treatment class. Each trial was assigned to a treatment class based on the mechanism of action of the experimental arm of the trial. Chemo = Chemotherapy, EGFR = Epidermal Growth Factor Receptor, VEGF = Vascular Endothelial Growth Factor, BSC = Best Supportive Care.

Treatment Class (Total Trial-Subgroups)	Treatment Arm Combination	Number of Trial-Subgroups
**Chemo (11)**	Chemo + BSC vs. Placebo + BSC	2
	Chemo vs. Chemo	5
	Chemo vs. Placebo	2
	Chemo vs. VEGF	1
	Chemo vs. VEGF + Chemo	1
**EGFR (6)**	EGFR + BSC vs. BSC	3
	EGFR vs. BSC	1
	EGFR vs. EGFR	1
	EGFR vs. EGFR + Chemo	1
**EGFR + Chemo (31)**	EGFR + Chemo vs. Chemo	18
	EGFR + Chemo vs. EGFR	1
	EGFR + Chemo vs. EGFR + Chemo	7
	EGFR + Chemo vs. VEGF + Chemo	5
**VEGF (8)**	VEGF + BSC vs. Placebo + BSC	2
	VEGF vs. Placebo	4
	VEGF vs. VEGF + Chemo	2
**VEGF + Chemo (19)**	VEGF + Chemo vs. Chemo	14
	VEGF + Chemo vs. EGFR + Chemo	1
	VEGF + Chemo vs. Observation	2
	VEGF + Chemo vs. VEGF + Chemo	2
**VEGF +** **EGFR (4)**	VEGF + EGFR vs. EGFR	1
	VEGF + EGFR vs. VEGF	3
**VEGF + EGFR** **+ Chemo (6)**	VEGF + EGFR + Chemo vs. Chemo	2
	VEGF + EGFR + Chemo vs. VEGF + Chemo	4
**Studies that could** **not be separated** **into a treatment** **class (15)**	ANG1/2/TIE2 + Chemo vs. Chemo	2
	BSC vs. EGFR + BSC	2
	Chemo ± VEGF vs. Chemo ± VEGF	2
	C-met + Chemo vs. Chemo	2
	EGFR + CD51 + Chemo vs. EGFR + Chemo	1
	EGFR + HGF vs. EGFR	1
	EGFR + IGF-1 + Chemo vs. EGFR + Chemo	2
	HER3 + Chemo vs. EGFR + Chemo	1
	LOXL2 + Chemo vs. Chemo	1
	TRAIL + Chemo vs. Chemo	1

**Table 2:** Full list of trials included for each analysis.

			Surrogate Relationship		
**Treatment** **Class**	**Study ID**	**KRAS Status**	**PFS for OS**	**TR for PFS**	**TR for OS**
**Chemo**	Hagman 2016 [19]	MT	✓	X	X
	Lenz 2017 [20]	MT	✓	✓	✓
	Maughan 2011 [21]	WT	✓	✓	✓
	Mayer 2015 [22]	WT	✓	X	X
		MT	✓	X	X
	Passardi 2017 [23]	WT	✓	✓	✓
	Reinacher-Schick 2012 [24]	WT	✓	X	X
	Richman 2009 [25]	WT	✓	X	X
		MT	✓	X	X
	Yoshino 2012 [26]	MT	✓	✓	✓
		WT	✓	✓	✓
**EGFR**	Aranda 2018 [27]	WT	✓	X	X
	Harbison 2013 [28]	MT	✓	X	X
		WT	✓	X	X
	Kim 2018 [29]	WT	✓	✓	✓
	Montagut 2018 [30]	WT	X	X	✓
	Price 2016 [31]	WT	✓	✓	✓
**EGFR +** **Chemo**	Bokemeyer 20011 [32]	MT	✓	✓	✓
		WT	✓	✓	✓
	Bridgewater 2017 [33]	WT	✓	✓	✓
	Brodowicz 2013 [34]	WT	✓	✓	✓
	Carrato 2017 [35]	WT	✓	✓	✓
	Cascinu 2017 [36]	WT	✓	✓	✓
	Ciardiello 2016 [37]	WT	✓	✓	✓
	Douillard 2014 [38]	MT	✓	✓	✓
		WT	✓	✓	✓
	Douillard 2014 (2) [39]	MT	✓	✓	✓
		WT	✓	✓	✓
	Hara 2017 [40]	WT	✓	✓	✓
	Hecht 2015 [41]	WT	✓	✓	✓
	Heinemann 2014 [42]	WT	✓	✓	✓
	Maughan 2014 [43]	WT	X	✓	X
	Munemoto 2019 [44]	WT	✓	✓	✓
	Peeters 2010 [45]	MT	✓	✓	✓
		WT	✓	✓	✓
	Peeters 2014 [46]	MT	✓	✓	✓
		WT	✓	✓	✓
	Qin 2018 [47]	WT	✓	X	X
	Schwartzberg 2014 [48]	WT	✓	✓	✓
	Seymour 2013 [49]	WT	✓	✓	✓
	Shapiro 2018 [50]	WT	✓	✓	✓
	Shitara 2016 [51]	WT	✓	✓	✓
	Tveit 2012 [52]	MT	✓	✓	✓
		WT	✓	✓	✓
	VanCutsem 2011 [53]	MT	✓	✓	✓
		WT	✓	✓	✓
	Venook 2017 [54]	WT	✓	X	X
	Ye 2013 [55]	WT	✓	✓	✓
**VEGF**	Garcia 2011 [56]	MT	✓	✓	✓
		WT	✓	✓	✓
	Li 2015 [57]	MT	✓	X	X
		WT	✓	X	X
	Li 2018 [58]	MT	✓	X	X
		WT	✓	X	X
	Tabernero 2012 [59]	WT	✓	X	X
		MT	✓	X	X
**VEGF +** **Chemo**	Bennouna 2013 [60]	MT	✓	✓	✓
		WT	✓	✓	✓
	Bennouna 2018 [61]	WT	✓	✓	✓
	Goey 2016 [62]	MT	✓	X	X
		WT	✓	X	X
	Hurwitz 2009 [63]	MT	✓	✓	✓
		WT	✓	✓	✓
	Nakayama 2018 [64]	MT	✓	✓	✓
		WT	✓	✓	✓
	Price 2011 [65]	WT	✓	X	X
		MT	✓	X	X
	Smith 2013 [66]	MT	✓	X	X
		WT	✓	X	X
	Tabernero 2013 [67]	MT	✓	X	X
		WT	✓	X	X
	Tabernero 2015 [68]	MT	✓	X	X
		WT	✓	X	X
	Van Cutsem 2012 [69]	WT	✓	X	X
		MT	✓	X	X
**VEGF +** **EGFR**	Hagman 2016 [19]	WT	✓	X	X
	Siu 2013 [70]	WT	✓	✓	✓
	Tournigand 2015 [71]	MT	✓	✓	✓
		WT	✓	✓	✓
**VEGF +** **EGFR +** **Chemo**	Liu 2015 [72]	MT	✓	✓	✓
		WT	✓	✓	✓
	PACCE (Iri-CT) [73]	MT	✓	✓	✓
		WT	✓	✓	✓
	PACCE (Ox-CT) [73]	MT	✓	✓	✓
		WT	✓	✓	✓
**Not assigned**	Bendell 2017 [74]	WT	✓	X	X
		MT	✓	X	X
	Cohn 2013 [75]	MT	✓	✓	✓
	Elez 2015 [76]	WT	✓	X	X
	Hecht 2017 [77]	MT	✓	✓	✓
	Hill 2018 [78]	WT	✓	✓	✓
	Peeters 2013 [79]	MT	✓	X	X
		WT	✓	X	X
	Peeters 2013 (2) [80]	MT	X	✓	X
		WT	X	✓	X
	Sclafani 2015 [81]	WT	✓	✓	✓
	VanCutsem 2014 [82]	WT	✓	✓	✓
	Watkins 2012 [83]	WT	✓	X	X
	Xu 2018 [84]	MT	✓	X	X
		WT	✓	X	X

**Figure A1:**
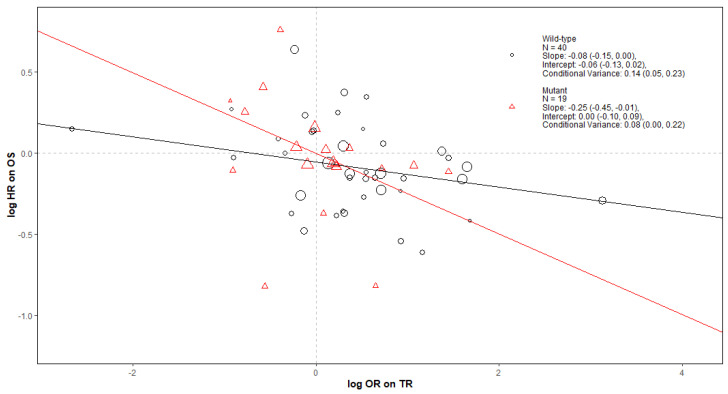
Bubble plot of TR as a surrogate for OS in RCTs of KRAS WT and MT mCRC. The Slope, Intercept and Conditional variance are mean estimates with 95% Credible Intervals obtained from Daniels and Hughes model. N represents the number of trial-subgroups.

**Figure A2:**
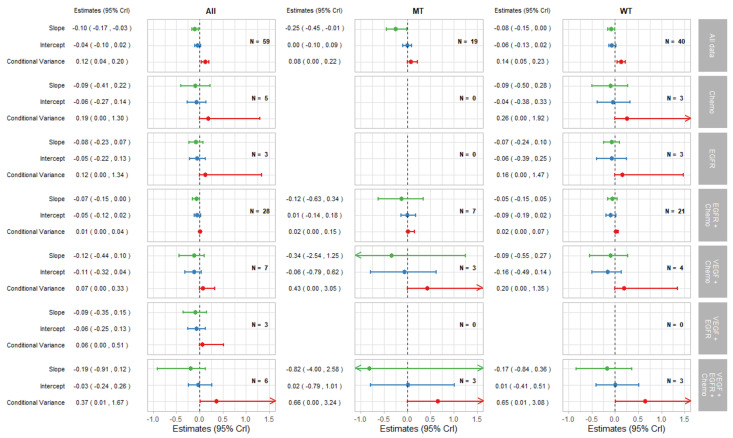
Forest plot of estimates of slope (green), intercept (blue), and conditional variance (red) for TR as a surrogate for OS. N represents the number of trial-subgroups.

## Data Availability

The data presented in this study are obtained from publicly available sources, all of which are listed in Table 2 and the references section.

## Appendix A. Further Methods

### Appendix A.1. Trial Identification: Search Strategies

All searches carried out on 3 April 2020.

#### Appendix A.1.1. Cochrane Central Register of Controlled Trials (CENTRAL) in the Cochrane Library

#1	MeSH descriptor: [Immunotherapy] explode all trees	7760
#2	MeSH descriptor: [Angiogenesis Modulating Agents] explode all trees	1132
#3	MeSH descriptor: [Antibodies, Monoclonal] explode all trees	12,528
#4	MeSH descriptor: [Antineoplastic Agents] explode all trees	11,805
#5	MeSH descriptor: [Drug Therapy] explode all trees	137,825
#6	MeSH descriptor: [Antigens, Neoplasm] explode all trees	2149
#7	chemotherap*	78,829
#8	immunotherap*	10,758
#9	(antitum?r or "anti tum?r" or anti-tum?r)	5131
#10	inhibitor	52,242
#11	cytotoxic	4112
#12	cytostatic	501
#13	(target* next (treatment or agent or therapy or administration or drug))	3166
#14	(hormone* next (treatment or agent or therapy or administration or drug))	5148
#15	(drug next (treatment or agent or therapy or administration))	405,830
#16	(antineoplas* or anti neoplas* or anti-neoplas*)	29,472
#17	(anticancer* or anti cancer* or anti-cancer*)	14,743
#18	(antiangiogen* or anti-angiogen* or anti angiogen*)	2049
#19	infusion	58,181
#20	immune response	15,989
#21	(pharmacologic* next (treatment or agent or therapy or administration))	5686
#22	antigen	19,362
#23	#1 or #2 or #3 or #4 or #5 or #6 or #7 or #8 or #9 or #10 or #11 or #12 or #13 or #14 or #15 or #16 or #17 or #18 or #19 or #20 or #21 or #22	556,231
#24	MeSH descriptor: [Colorectal Neoplasms] explode all trees	7912
#25	MeSH descriptor: [Neoplasm Metastasis] explode all trees	4995
#26	(((colorect* or colon* or rect* or anal* or intestin* or bowel* or sigmoid*) near/3 (carcinom* or neoplas* or adenocarcinom* or cancer* or tumor* or tumour* or sarcom* or adenoma*)) near/4 (metastas* or metastatic* or micrometastas* or micrometastatic* or advance* or "stage IV" or "stage 4" or "stage four" or irresectable or unresectable or palliati*))	7801
#27	(CRC near/4 (metastas* or metastatic* or micrometastas* ir micrometastatic* or advance* or "stage IV" or "stage 4" or "stage four" or irresectable or unresectable or palliati*))	1783
#28	aCRC or mCRC	1662
#29	#24 and #25	706
#30	#26 or #27 or #28 or #29	8148
#31	#30 and #23 with Publication Year from 2003 to 2020, in Trials	5047

#### Appendix A.1.2. Ovid MEDLINE

1	exp Immunotherapy/	271,523
2	exp Angiogenesis Modulating Agents/	62,615
3	exp Antibodies, Monoclonal/	232,456
4	exp Antineoplastic Agents/	1,085,139
5	exp Drug Therapy/	1,341,564
6	exp Antigens, Neoplasm/	116,062
7	chemotherap*.mp.	477,700
8	immunotherap*.mp.	111,265
9	(antitum?r or “anti tum?r” or anti-tum?r).mp.	166,008
10	inhibitor.mp.	652,707
11	cytotoxic.mp.	190,318
12	cytostatic.mp.	13,996
13	(target* adj (treatment or agent or therapy or administration or drug)).mp.	65,873
14	(hormone* adj (treatment or agent or therapy or administration or drug)).mp.	22,054
15	(drug adj (treatment or agent or therapy or administration)).mp.	2,373,269
16	(antineoplas* or anti neoplas* or anti-neoplas*).mp.	499,852
17	(anticancer* or anti cancer* or anti-cancer*).mp.	120,875
18	(antiangiogen* or anti-angiogen* or anti angiogen*).mp.	25,384
19	infusion.mp.	226,908
20	immune response.mp.	155,377
21	(pharmacologic* adj (treatment or agent or therapy or administration)).mp.	28,789
22	antigen.mp.	628,662
23	1 or 2 or 3 or 4 or 5 or 6 or 7 or 8 or 9 or 10 or 11 or 12 or 13 or 14 or 15 or 16 or 17 or 18 or 19 or 20 or 21 or 22	5,271,893
24	randomized controlled trial.pt.	503,142
25	controlled clinical trial.pt.	93,595
26	randomized.ti,ab.	513,184
27	placebo.ti,ab.	212,175
28	clinical trials as topic.sh.	190,619
29	randomly.ti,ab.	331,224
30	trial.ti.	215,880
31	24 or 25 or 26 or 27 or 28 or 29 or 30	1,287,975
32	exp animals not humans.sh.	4,685,426
33	31 not 32	1,185,372
34	(((colorect* or colon* or rect* or anal* or anus* or intestin* or bowel* or sigmoid*) adj3 (carcinom* or neoplas* or adenocarcinom* or cancer* or tumor* or tumour* or sarcom* or adenoma*) adj4 (metastas* or metastatic* or micrometastas* or micrometastatic* or advance* or “stage IV” or “stage 4” or “stage four” or irresectable or unresectable or palliati*)) or "aCRC" or “mCRC”).mp.	40,955
35	(CRC adj4 (metastas* or metastatic* or micrometastas* or micrometastatic* or advance* or “stage IV” or “stage 4” or “stage four” or irresectable or unresectable or palliati*)).mp. [mp=title, abstract, original title, name of substance word, subject heading word, floating sub-heading word, keyword heading word, organism supplementary concept word, protocol supplementary concept word, rare disease supplementary concept word, unique identifier, synonyms]	5279
36	exp colorectal neoplasms/	197,894
37	exp neoplasm metastasis/	200,932
38	36 and 37	17,461
39	34 or 35 or 38	52,675
40	33 and 39	5701
41	23 and 40	4736
42	limit 41 to yr="2003 -Current"	3413

#### Appendix A.1.3. Ovid EMBASE

1	exp drug therapy/	2,759,981
2	exp immunotherapy/	212,046
3	exp drug activity/	2,336,899
4	exp monoclonal antibody/	563,094
5	exp cancer therapy/	802,327
6	exp antigen/	1,561,070
7	chemotherap*.mp.	843,036
8	immunotherap*.mp.	197,910
9	(antitum?r or “anti tum?r” or anti-tum?r).mp.	169,575
10	inhibitor.mp.	1,419,535
11	cytotoxic.mp.	273,532
12	cytostatic.mp.	21,483
13	(target* adj (treatment or agent or therapy or administration or drug)).mp.	93,739
14	(hormone* adj (treatment or agent or therapy or administration or drug)).mp.	47,449
15	(drug adj (treatment or agent or therapy or administration)).mp.	5,633,269
16	(antineoplas* or anti neoplas* or anti-neoplas*).mp.	460,857
17	(anticancer* or anti cancer* or anti-cancer*).mp.	163,990
18	(antiangiogen* or anti-angiogen* or anti angiogen*).mp.	45,610
19	infusion.mp.	368,696
20	immune response.mp.	356,585
21	(pharmacologic* adj (treatment or agent or therapy or administration)).mp.	44,297
22	antigen.mp.	1,339,922
23	1 or 2 or 3 or 4 or 5 or 6 or 7 or 8 or 9 or 10 or 11 or 12 or 13 or 14 or 15 or 16 or 17 or 18 or 19 or 20 or 21 or 22	9,964,936
24	random*.ab,ti. or placebo*.de,ab,ti. or (double adj1 blind*).ab,ti.	1,772,713
25	(exp animal/ or exp nonhuman/ or exp animal experiment/ or exp experimental animal/) not exp human/	6,440,460
26	24 not 25	1,566,673
27	exp “metastatic colorectal cancer”/	10,749
28	(((colorect* or colon* or rect* or anal* or anus* or intestin* or bowel* or sigmoid*) adj3 (carcinom* or neoplas* or adenocarcinom* or cancer* or tumor* or tumour* or sarcom* or adenoma*) adj4 (metastas* or metastatic* or micrometastas* or micrometastatic* or advance* or “stage IV” or “stage 4” or “stage four” or irresectable or unresectable or palliati*)) or “aCRC” or “mCRC”).mp.	67,297
29	(CRC adj4 (metastas* or metastatic* or micrometastas* or micrometastatic* or advance* or “stage IV” or “stage 4” or “stage four” or irresectable or unresectable or palliati*)).mp.	9236
30	27 or 28 or 29	70,548
31	23 and 26 and 30	7155
32	limit 31 to yr = “2003 -Current”	6380

### Appendix A.2. Data Extraction: Definitions of Treatment Effects

PFS was defined as time taken from randomisation or from start of treatment, until tumour progression or death (of any cause). OS was defined as time from randomisation or start of treatment until death from any cause. TR was defined as patients achieving complete or partial response at the time point specified in each RCT. For solid tumours, a partial or complete response was defined as a decrease in the tumour size usually with reference to Response Evaluation Criteria in Solid Tumours (RECIST) [88] or World Health Organisation (WHO) criteria [89] or in some cases individual trial criteria. The numbers of responders and total numbers of participants were recorded to estimate treatment effects on TR using odds ratios (ORs).

### Appendix A.3. Statistical Methods

#### Appendix A.3.1. Cross Validation

Take-one-out cross-validation procedure was carried out to investigate the predictive value of each surrogate endpoint. The proportion of the observed effect estimates that fall within the predicted interval, the absolute difference of means of the observed and predicted effects, and the ratio of the widths between the observed and predicted intervals were calculated for each model. By chance, it is expected that around 5% of observed estimates may fall outside of the 95% predictive interval.

#### Appendix A.3.2. Further Statistical Methods

Within-study correlation is needed for each trial to populate the model, however this is rarely reported for RCTs. Within-study correlations between the treatment effects on PFS and OS, between the effects on TR and PFS, and between TR and OS were provided by collaborators at Roche, obtained from four RCTs for which individual patient data were available. Average correlations across the trials reported for each of the surrogate relationships were used in the analysis assuming the same correlation across trials.

## Appendix B. Further Results

### Appendix B.1. Results and Conclusions for TR as a Surrogate for OS

#### Appendix B.1.1. Results

TR as a surrogate for OS proved to be a weak surrogate endpoint (Figure A1 and Figure A2). This is due to the conditional variances being relatively large.

Exploring the results by KRAS status for TR-OS, there was no particular difference in surrogate relationship between KRAS subgroups and overall cohort of patients, as seen in Figure A1, and top row of Figure A2.

For the TR-OS pair of outcomes by KRAS status and treatment class in Figure A2, the surrogate relationship was moderate only for the EGFR + Chemo treatment class when both MT and WT trial-subgroups are included. When looking at either KRAS MT or WT trial-subgroups only, the surrogate relationships were weak within each treatment class; however, the data for these analyses were limited.

#### Appendix B.1.2. Conclusions

Our results indicated a sub-optimal surrogate relationship between the treatment effects on TR and the effects on OS for mCRC, reaching a similar conclusion as Ciani et al. [8]. This was the case in all three analyses; of all data and the KRAS status subgropus.There was some evidence from the results that the surrogacy patterns may vary according to the mechanism of action, with relatively strong surrogate relationship for EGFR+Chemo therapies.

Fewer trials reported TR than PFS and OS results, which led to more uncertainty around the estimates produced from the analyses for TR. In addition, TR was defined at a specific time point and using a particular criteria, e.g., RECIST [88], WHO criteria [89] or individual trial criteria, which varied between RCTs and could account for the increased between-studies heterogeneity of the treatment effect on TR and therefore potentially weaker surrogate relationship. Further analysis could be undertaken to explore how TR was defined within each trial and whether this affects the strength of the surrogate relationship.

In summary the results from this investigation suggest that TR is not a strong surrogate endpoint for OS when evaluating pharmacological therapies for mCRC patients overall, but could potentially be used as a surrogate endpoint when evaluating EGFR+Chemo therapies. The overall conclusions also hold for subgroups of population by KRAS status, but there was no evidence of the importance of the mechanism of action, potentially due to the limited data.

### Appendix B.2. Cross Validation and Predictions

Table A1 shows the results of the cross validation procedure for PFS as a surrogate for OS. The Daniels and Hughes method showed a large coverage in terms of the proportion of the 95% predicted intervals containing the observed effect estimate. For the Hierarchical model, taking into account treatment class, the cross validation for the KRAS MT and WT trial-subgroups resulted in all 95% prediction intervals including the observed estimates of the effect on OS, whereas cross validation using the Daniels and Hughes method had 3.61% of the predicted intervals not including the observed estimates of the treatment effect on OS.

**Table A1:** Summary of cross validation results for all analyses for PFS as a surrogate for OS.

Trial-Subgroups	All		MT		WT	
Model	Daniels and Hughes	Hierarchical	Daniels and Hughes	Hierarchical	Daniels and Hughes	Hierarchical
Percentage of observed effect estimates within 95% predicted interval	94.79%	96.39%	94.29%	100.00%	95.08%	100.00%
Average absolute difference between the observed and predicted effect estimates	0.16	0.16	0.17	0.20	0.15	0.16
Average ratio of the width of intervals between the predicted and observer treatment effects	1.14	1.51	1.13	2.14	1.21	2.02

There was 0.16 average absolute difference between the observed effect estimate and the predicted effect for OS for each trial from both the Daniels and Hughes model and the Hierarchical model including all trial-subgroups and also for KRAS WT trial-subgroups when using the hierarchical model, with a slightly smaller average of 0.15 when using Daniels and Hughes model. For KRAS MT, the average discrepancy was slightly higher, 0.20 from the hierarchical model and 0.17 from Daniels and Hughes model.

The ratios of the width of intervals indicate that the predictions obtained from the hierarchical model were obtained with larger uncertainty compared to the predictions from the Daniels and Hughes model of all data on all treatment classes combined. This is likely due to the predictions in the treatment classes with small number of studies being obtained with large uncertainty from the hierarchical model.
